# Pennsylvania State Board of Veterinary Examiners

**Published:** 1895-11

**Authors:** 


### PENNSYLVANIA'S STATE BOARD OF VETERINARY EXAMINERS.

Governor Hastings, on October 11th, made the following appointments to constitute the State Board of Veterinary Medical Examiners, their terms to compute from the first Monday in September, 1895: W. Horace Hoskins, Philadelphia, graduate of the American Veterinary College, for three years; S. J. J. Harger, of Philadelphia, graduate of and Professor in the Veterinary Department of the University of Pennsylvania, for three years; J. C. McNeil, of Pittsburg, and Harry Walter, of Wilkesbarre, each for two years, both graduates of the Veterinary Department of the University of Pennsylvania.; and J. W. Sallade, of Pottsville, graduate of the Toronto Veterinary College.

---

Judge Stewart, of Pennsylvania, recently decided that the visiting lists, so very popular among veterinarians and physicians, do not constitute books of original entry, but are simply memoranda, and will not be recognized as evidence in court in proof of claim for professional services.

The Society for the Prevention of Cruelty to Animals in Richmond, Va., has just obtained a decision in their favor, where an arrest of a blacksmith for burning out lampass was made on the ground of cruelty. A few more such movements would do away with this ignorant, barbarous treatment of a condition which is generally symptomatic only.

The recent deaths and severe illness of a large number of persons at Sabula, Iowa, are now believed to be the result of trichinosis. The time has now arrived when official inspection should precede the sale and consumption of all our meat-products.

The skull of a monkey recently found in Wyoming by Prof. Wortman is supposed to be about one million years old, and to represent the Eocene period. It is believed that further investigation will determine that it is the missing link, and to represent a midway development between the monkey's cranium and man's.

The Department of Agriculture will shortly issue a bulletin advocating the culture of cork-trees in this country, especially in the South and in California.

The English are now using, as a substitute for iodide of potash, an iodinated preparation of lime, and claim equally as effective results from its use in actinomycosis.

